# MET Receptor Tyrosine Kinase Regulates the Expression of Co-Stimulatory and Co-Inhibitory Molecules in Tumor Cells and Contributes to PD-L1-Mediated Suppression of Immune Cell Function

**DOI:** 10.3390/ijms20174287

**Published:** 2019-09-01

**Authors:** Hyun Kyung Ahn, Sehui Kim, Dohee Kwon, Jaemoon Koh, Young A. Kim, Kwangsoo Kim, Doo Hyun Chung, Yoon Kyung Jeon

**Affiliations:** 1 Cancer Research Institute, Seoul National University, Seoul 03080, Korea; 2 Department of Pathology, Seoul National University College of Medicine, Seoul 03080, Korea; 3 Department of Biomedical Sciences, Seoul National University College of Medicine, Seoul 03080, Korea; 4 Department of Pathology, Seoul Metropolitan Government Seoul National University Boramae Medical Center, Seoul 03080, Korea; 5 Division of Clinical Bioinformatics, Biomedical Research Institute, Seoul National University Hospital, Seoul 03080, Korea

**Keywords:** MET, immune evasion, immune checkpoint, co-stimulatory molecules, co-inhibitory molecules, PD-1/PD-L1 pathway, PD-L1, cancer immunotherapy, lung cancer

## Abstract

The MET tyrosine receptor kinase is essential for embryonic development and tissue regeneration by promoting cell survival, proliferation, migration, and angiogenesis. It also contributes to tumor development and progression through diverse mechanisms. Using human cancer cell lines, including Hs746T (*MET*-mutated/amplified), H596 (*MET*-mutated), and H1993 (*MET*-amplified) cells, as well as BEAS-2B bronchial epithelial cells, we investigated whether MET is involved in the regulation of immune checkpoint pathways. In a microarray analysis, MET suppression using a MET inhibitor or siRNAs up-regulated co-stimulatory molecules, including 4-1BBL, OX40L, and CD70, and down-regulated co-inhibitory molecules, especially PD-L1, as validated by measuring total/surface protein levels in Hs746T and H1993 cells. MET activation by HGF consistently increased PD-L1 expression in H596 and BEAS-2B cells. Co-culture of human peripheral blood mononuclear cells with Hs746T cells suppressed interferon-γ production by the immune cells, which was restored by MET inhibition or PD-L1 blockade. A significant positive correlation between MET and PD-L1 expression in lung cancer was determined in an analysis based on The Cancer Genome Atlas (TCGA) and in an immunohistochemistry study. The former also showed an association of MET overexpression in a PD-L1^high^ tumor with the decreased expressions of T-cell effector molecules. In summary, our results point to a role for MET overexpression/activation in the immune escape of tumors by PD-L1 up-regulation. MET-targeted-therapy combined with immunotherapy may therefore be an effective treatment strategy in patients with MET-dependent cancer.

## 1. Introduction

MET is a receptor tyrosine kinase that plays an important role in cancer development and progression [1]. MET genetic alterations are found in many types of tumors and are associated with their aggressive behavior as well as their resistance to therapy [2]. Resistance to EGFR tyrosine kinase inhibitor (TKI) in non-small cell lung cancer (NSCLC) has been attributed to *MET* amplification [3]. MET protein overexpression occurs in 22–75% of NSCLCs and correlates with phospho-MET (p-MET) expression, suggesting a relationship between MET overexpression and activation [4,5]. Among the *MET* mutations described thus far, a somatic mutation at or around the exon 14 splice junction that leads to deletion of the exon and, thus, to a protein lacking the juxtamembrane domain has been implicated in oncogenic MET activation [6,7]. The juxtamembrane domain is the site of c-Cbl binding, required for the degradation of MET following its signaling activation via binding to hepatocyte growth factor (HGF; a MET ligand). The *MET* exon 14 skipping mutation causes impaired c-Cbl-mediated degradation of MET and therefore sustains MET activation [6,7]. Despite the heterozygosity of this mutation at the DNA level, the truncated form of MET is much more abundant than the wild-type [6,7]. The *MET* exon 14 skipping mutation is frequently observed in pulmonary sarcomatoid carcinoma (the most aggressive entity of NSCLC) [8,9,10,11]. Based on these observations, MET is considered a promising therapeutic target in NSCLC [1,2].

Immune checkpoint pathways refer to a variety of inhibitory interactions between T cells and target cells, including antigen-presenting cells and cancer cells. Examples include the programmed cell death-1 (PD-1)/programmed cell death-1-ligands (PD-Ls) and CTLA-4/CD80 pathways [12]. Engagement of PD-1 expressed on T cells by PD-Ls on antigen-presenting cells and tumor cells results in the suppression of T-cell proliferation and function, whereas PD-1/PD-L1 blockades restore effector T-cell function and anti-tumor immune responses [13]. Recently, PD-1 and PD-L1 blockades have been introduced as a novel therapeutic strategy for cancer therapy [13]. However, not all patients clinically benefit from this form of immune checkpoint blockade, such that other targets and strategies that enhance the efficacy of this approach to treatment are needed [14,15].

PD-L1 expression in tumor cells and immune cells is associated with the efficacy of PD-1/PD-L1 blockades in cancer patients and thus serves as a predictive biomarker [13,16]. The U.S. Food and Drug Administration has approved PD-L1 immunohistochemistry (IHC) as a companion diagnostic for NSCLC, gastric, or gastroesophageal junction adenocarcinoma, cervical cancer, urothelial cancer, squamous cell carcinoma of the head and neck and esophagus, and breast cancer. PD-L1 expression can be induced by endogenous oncogenic signaling in tumor cells or by exogenous cytokines (e.g., interferon-γ, IFNγ) secreted from immune cells [12]. We previously demonstrated a positive correlation between MET and PD-L1 expression in lung cancer [17,18]. While MET contributes to tumor progression and aggressiveness by diverse mechanisms [19], its role in the regulation of the tumor immune response is unclear. In this study we asked whether MET is involved in the regulation of immune checkpoint pathways and immune cell function and then validated our findings by analyzing tumor tissues from patients and a public tumor database. Our study showed the following: (1) MET activation up-regulates co-inhibitory molecules (particularly PD-L1) and down-regulates co-stimulatory molecules; (2) MET inhibition in tumor cells enhances the function of co-cultured immune cells; (3) MET expression by the tumors of cancer patients, including those with NSCLC, and in cell lines positively correlates with that of PD-L1; and (4) MET overexpression is related to immunosuppressive features in the tumor microenvironment of PD-L1^high^ NSCLC. Taken together, these results suggest that MET may be involved in tumor immune evasion. Combined MET-targeted therapy and immunotherapy may therefore be an effective strategy in the treatment of several forms of cancer.

## 2. Results

### 2.1. MET Inhibition or Knockdown in Hs746T Cells Causes Changes in the Expression of Immune-Response-Related Genes

Lung adenocarcinoma cell lines, including H596 (harboring a *MET* exon 14 skipping mutation) and H1993 (harboring a *MET* amplification), and a gastric carcinoma cell line, Hs746T (harboring both a *MET* exon 14 skipping mutation and *MET* amplification), were used in this study. In H1993 cells and Hs746T cells, p-MET expression was up-regulated in the absence of HGF (Supplementary Figure S1) whereas in H596 cells HGF treatment resulted in prolonged p-MET expression/MET activation compared to cells carrying wild-type *MET* [6].

The regulation of gene expression by MET was further examined in Hs746T cells, which, among the cell lines included in this study, had the highest basal-level expression of MET, p-MET, and PD-L1 and showed a marked decrease in the levels of MET and p-MET after MET inhibitor treatment or siRNA transfection. These cells were treated with the MET inhibitor PHA665752 or transfected with MET siRNA1, the most effective siRNA (Supplementary Figure S2A), and submitted to microarray analysis (GEO accession number; GSE135976). Overall, 15.4% and 4.3% of total genes were significantly changed more than 2-fold by the MET inhibitor treatment and MET knockdown, respectively. The difference may be partly attributable to the different efficacies of MET inhibitor and siRNA in MET and p-MET down-regulation. p-MET was more effectively down-regulated by MET inhibitor (PHA665752) than by MET knockdown. Gene ontology (GO) analysis of the genes up- or down-regulated by MET suppression revealed their involvement in angiogenesis, apoptosis, cell cycle, cell migration, cell proliferation, DNA repair, extracellular matrix, and immune and inflammatory responses (Supplementary Figure S2B,C). Discrepancies in the GO analysis of cells treated with MET inhibitor versus MET knockdown can also be attributed to the different efficacies of MET suppression by PHA665752 and siRNA and to the unintended biological effect of both inhibitors.

**Figure 1:**
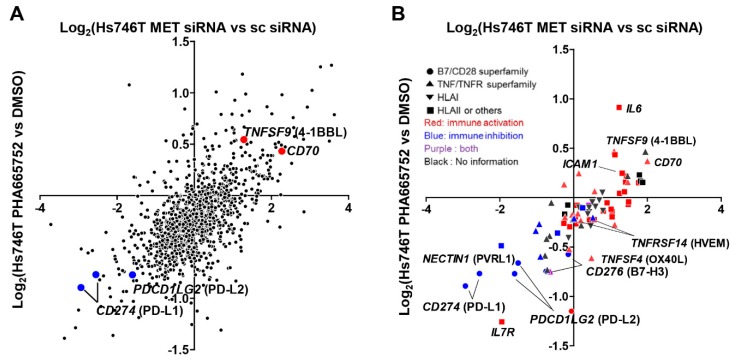
Changes in the immune-response-related gene expression of Hs746T cells after MET inhibition and knockdown. The changes in all immune-response-related genes (**A**) and in co-inhibitory and co-stimulatory molecules as well as HLA molecules (**B**) were plotted for cells treated with the MET inhibitor PHA665752 or transfected with MET siRNA (sc siRNA, scrambled siRNA).

Nonetheless, the changes in the expression of immune-related genes were well-correlated between cells treated with MET inhibitor and MET knockdown (Figure 1A). Plots of the genes belonging to the B7/CD28 and TNF/TNFR superfamilies and to the HLA family showed the up-regulation of co-stimulatory molecules, such as *TNFSF9* (4-1BB ligand, 4-1BBL), *CD70*, and *ICAM1*, and the down-regulation of co-inhibitory molecules, including *CD274* (PD-L1), *PDCD1LG2* (PD-L2), *PVRL1*, and *CD276* (B7-H3) following MET suppression (Figure 1B). These genes were therefore likely to be responsive to MET signaling.

### 2.2. MET Down-Regulates the Expression of Co-Stimulatory Molecules and Up-Regulates the Expression of Co-Inhibitory Molecules, Particularly PD-L1, in Tumor and Non-Neoplastic Bronchial Epithelial Cells

**Figure 2:**
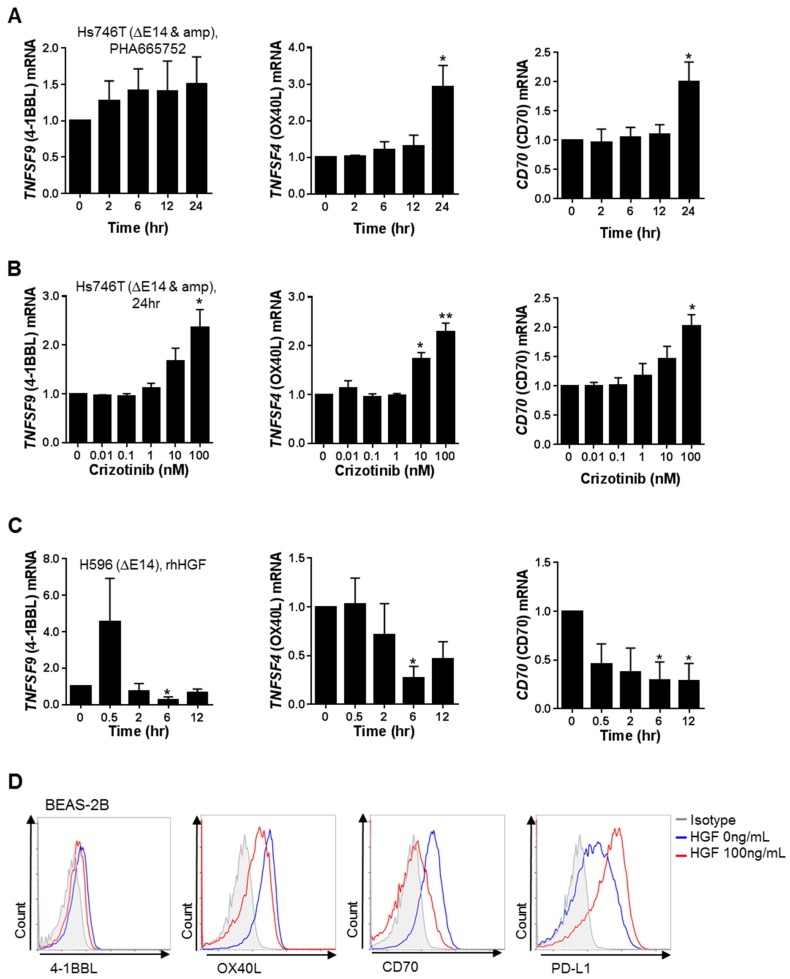
Changes in the expression of co-stimulatory molecules by MET signaling in human cancer cells and non-neoplastic bronchial epithelial cells. (**A**) Hs746T cells were treated with 400 nM of the MET inhibitor PHA665752 for the indicated times, after which the changes in *TNFSF9* (4-1BBL, 4-1BB ligand), *TNFSF4* (OX40L, OX40 ligand), and *CD70* mRNA expression were determined by qRT-PCR. (**B**) Hs746T cells were treated with the MET inhibitor crizotinib at the indicated concentrations for 24 h and the changes in *TNFSF9* (4-1BBL), *TNFSF4* (OX40L), and *CD70* mRNA expression were then determined by qRT-PCR. (**C**) H596 cells maintained in serum-free medium overnight were treated with 100 ng rhHGF/mL for the indicated times, after which the changes in *TNFSF9* (4-1BB ligand), *TNFSF4* (OX40 ligand), and *CD70* mRNA expression were determined by qRT-PCR. (**D**) BEAS-2B cells were incubated in serum-free medium for 6 h and then treated with rhHGF for 12 h. The surface expression of 4-1BBL, OX40L, CD70 and PD-L1 was analyzed by flow cytometry. Relative mRNA levels are presented as the fold-change compared to the control (2^−∆∆*C*t^). Data represent the mean ± SEM of at least three independent experiments. All *p* values were calculated using unpaired Student’s *t*-tests. * *p* < 0.05, ** *p* < 0.01.

Consistent with the microarray data, mRNA expression levels of the co-stimulatory molecules *TNFSF9* (4-1BBL), *TNFSF4* (OX40 ligand, OX40L), and *CD70* were significantly increased in Hs746T cells treated with the MET inhibitors PHA665752 or crizotinib (Figure 2A,B). To examine the effect of MET activation, H596 cells, which have a relatively low level of basal MET/p-MET expression, were treated with recombinant human hepatocyte growth factor (rhHGF) (Supplementary Figure S1). Reductions in the mRNA levels of the co-stimulatory molecules 4-1BBL, OX40L, and CD70 were clearly observed 24 h after MET inhibition or 6–12 h after MET activation (Figure 2A,C). This lag in the response time suggested that pathways/factors other than direct MET signaling are involved in the MET-mediated expression of co-stimulatory molecules. MET activation of non-neoplastic human bronchial epithelial cells (BEAS-2B) by rhHGF treatment led to the down-regulation of *TNFSF9* (4-1BBL), *TNFSF4* (OX40L), and *CD70* and to the up-regulation of *CD274* (PD-L1), at both the mRNA and surface protein levels (Supplementary Figure S3 and Figure 2D).

**Figure 3:**
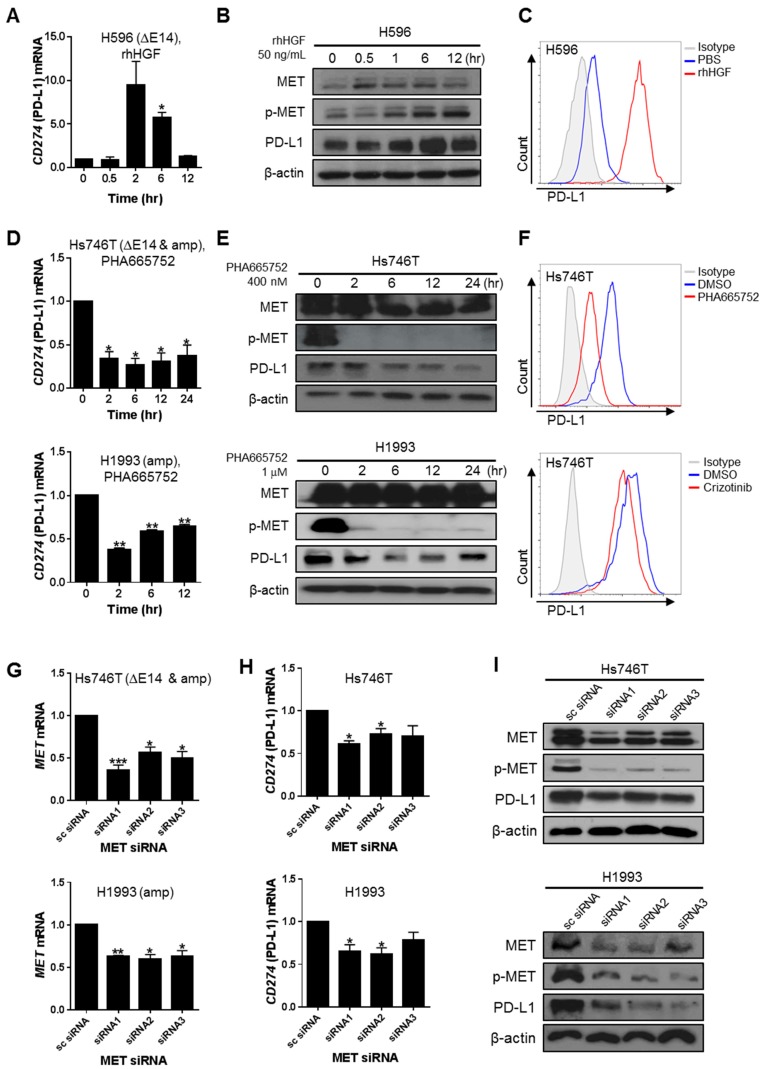
Changes in the expression of the co-inhibitory molecule PD-L1 by MET signaling activation in human cancer cells. (**A**,**B**) H596 cells were maintained in serum-free medium overnight and then treated with 50 ng rhHGF/mL for the indicated times. (**A**) Changes in *CD274* (PD-L1) mRNA levels were determined by qRT-PCR and are expressed as the fold-change compared to the control. (**B**) Changes in total MET, p-MET, and PD-L1 protein expression were analyzed by western blot. (**C**) The surface expression of PD-L1 was examined by flow cytometry in H596 cells treated for 24 h with 100 ng rhHGF/mL. (**D**,**E**) Changes in PD-L1 expression in Hs746T and H1993 cells treated for the indicated times with 400 nM and 1 µM PHA665752, respectively, were determined by qRT-PCR (**D**) and western blot for MET, p-MET, PD-L1, and β-actin (20 µg loading protein for Hs746T cells and 40 µg for H1993 cells) (**E**). (**F**) The surface expression of PD-L1 in Hs746T cells treated with 400 nM PHA665752 for 6 h or with 10 nM crizotinib for 24 h was analyzed by flow cytometry. (**G**–**I**) Hs746T and H1993 cells transfected with 100 nM MET-specific siRNA for 48 h were examined by qRT-PCR to determine changes in MET and *CD274* (PD-L1) mRNA expression (**G**,**H**) and by western blot to evaluate MET, p-MET, PD-L1 and β-actin expression (20 µg loading protein for Hs746T cells and 40 µg for H1993 cells) (**I**). Relative mRNA levels are presented as the fold-change compared to the control. Data represent the mean ± SEM or are representative of three independent experiments. Statistical significance was determined by unpaired Student’s *t*-tests. * *p* < 0.05, ** *p* < 0.01, *** *p* < 0.001.

In the microarray analysis, *CD274* (PD-L1) and *PDCD1LG2* (PD-L2) were the genes most significantly down-regulated by MET suppression. To determine whether a causal relationship existed between MET signaling and PD-L1 expression, p-MET^low^ H596 cells were stimulated with rhHGF and p-MET^high^ Hs746T and H1993 cells were treated with MET inhibitors or transfected with MET-specific siRNAs. MET activation of H596 cells by rhHGF enhanced PD-L1 expression at the mRNA, total protein, and surface expression levels (Figure 3A–C). Upon MET inhibition by PHA665752 or crizotinib, PD-L1 expression was significantly reduced at all three levels in both Hs746T and H1993 cells, whereas PD-L2 expression changed only slightly (Figure 3D–F and Supplementary Figure S4A–C). MET knockdown using siRNAs concomitantly down-regulated PD-L1 transcription and protein expression (Figure 3G–I and Supplementary Figure S4D). The MET-mediated down-regulation of co-stimulatory molecules and up-regulation of co-inhibitory molecules, particularly PD-L1, in cancer cells suggests that MET may contribute to the immune evasion of cancer cells.

### 2.3. MET-Overexpressing Cancer Cells Suppress the Function of Immune Cells

**Figure 4:**
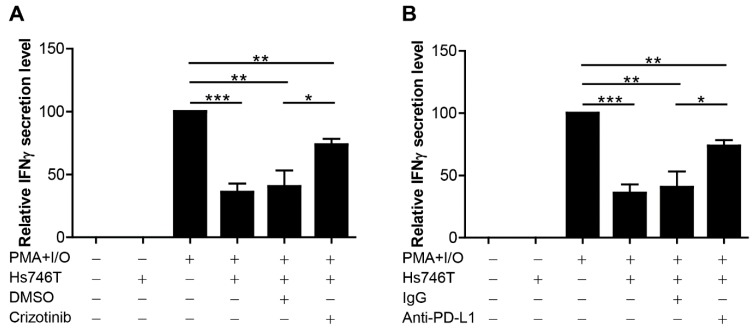
Co-culture of peripheral blood mononuclear cells (PBMCs) and PD-L1-expressing Hs746T cells. PBMCs were stimulated with 50 ng PMA/mL and 1 µg ionomycin/mL for 8 h. Hs746T cells were pre-treated or not with 10 nM crizotinib for 24 h (**A**) or with 40 µg anti-PD-L1 blocking antibody/mL for 12 h (**B**) and then co-cultured with the stimulated PBMCs for an additional 16 h. IFNγ production was determined by ELISA (enzyme-linked immunosorbent assay). Relative levels are presented as the fold-change compared to the control. Data are presented as the mean ± SEM of at least three independent experiments. All *p* values were calculated using unpaired Student’s *t*-tests. * *p* < 0.05, ** *p* < 0.01, *** *p* < 0.001.

To investigate the potential role of MET expressed by tumor cells in immune cell suppression, an in vitro co-culture system was developed using human peripheral blood mononuclear cells (PBMCs) and MET^high^/PD-L1^high^ Hs746T cells, as shown in Supplementary Figure S5. Increases in PD-1 levels on T cells (Supplementary Figure S5) and in IFNγ secretion were observed in PBMCs stimulated with PMA and ionomycin (Figure 4). Co-culture of stimulated PBMCs with Hs746T cells resulted in a significant inhibition of IFNγ secretion (Figure 4 and Supplementary Figure S6), which was partly restored by the addition of the MET inhibitor (crizotinib) (Figure 4A) and PD-L1 blocking antibody (Figure 4B). These data suggest that Hs746T cells suppress immune cell function via the MET pathway and PD-L1 expression.

### 2.4. Correlations between MET and PD-1 Ligand Expression in Human Cancer Tissues and Cell Lines

**Figure 5:**
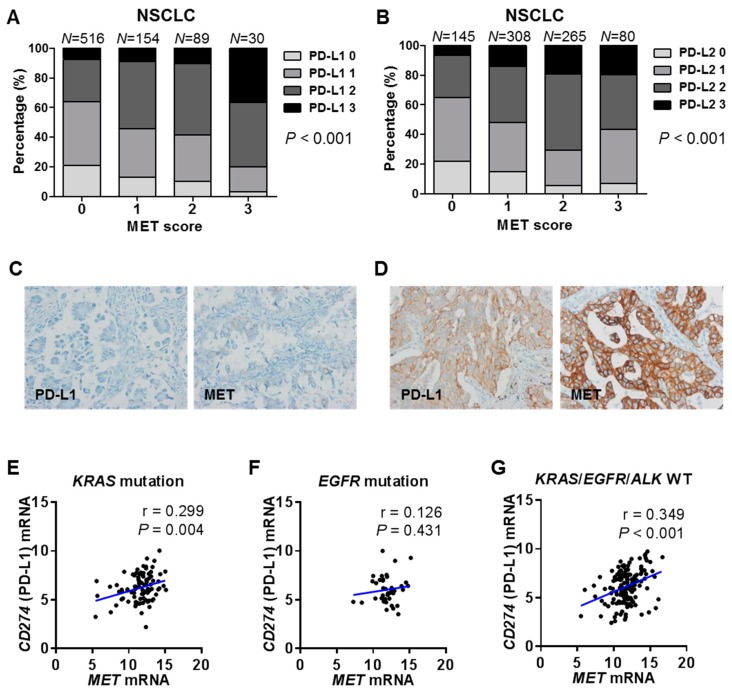
MET expression positively correlates with PD-L1 expression in human lung cancer tissues. (**A**,**B**) Comparative immunohistochemistry (IHC) analysis of MET and PD-L1 and PD-L2 expression in NSCLC tissues. (**C**,**D**) Representative IHC images for PD-L1 and MET in a patient with lung cancer lacking PD-L1 and MET expression (**C**) and in a patient with lung cancer characterized by both PD-L1 and MET expression (**D**) (original magnification, ×400). (**E**–**G**) The correlation between *CD274* (PD-L1) and *MET* mRNA levels was analyzed in lung adenocarcinoma carrying a *KRAS* mutation (*n* = 93), *EGFR* mutation (*n* = 41), and no *KRAS* mutation/*EGFR* mutation/*ALK* translocation (*n* = 152). The lung adenocarcinoma data were obtained from the TCGA and analyzed using a Pearson correlation analysis.

The potential role of MET in PD-L1 overexpression in human cancers was further examined by IHC of the NSCLC tissues from patients as well as by referencing the Cancer Cell Line Encyclopedia (CCLE) for pan-cancer cell lines and the TCGA for pan-cancers. In NSCLC tissues, IHC showed a significant positive correlation between MET and PD-L1 or PD-L2 expression by tumor cells (*p* < 0.001 for both), with a more consistent positive correlation of MET with PD-L1 than with PD-L2 (Figure 5A–D). An analysis of 14 cancer types (*n* = 5470) from the TCGA showed that the correlations between MET and PD-L1 or PD-L2 varied depending on the cancer type (Table 1). Of note, strong positive correlations were observed between MET and PD-L1 in lung adenocarcinoma and bladder cancer (r = 0.401, *p* < 0.001 and r = 0.385, *p* < 0.001, respectively) and between MET and PD-L2 in breast cancer and bladder cancer (r = 0.383, *p* < 0.001 and r = 0.355, *p* < 0.001, respectively). In stomach cancer, MET correlated weakly with PD-L1 but not PD-L2 (r = 0.137, *p* = 0.005 and r = 0.015, *p* = 0.754, respectively). In particular, a significant positive correlation between MET and PD-L1 expression was consistently observed in NSCLC tissues with either a *KRAS* mutation (*n* = 93) (r = 0.299, *p* = 0.004) or without *KRAS* mutation/*EGFR* mutation/*ALK* translocation (*n* = 152) (r = 0.349, *p* < 0.001), although a statistically significant positive correlation was not seen in those with an *EGFR* mutation (*n* = 41) (Figure 5E–G). The correlation between PD-L1 and MET was evaluated using the CCLE mRNA database, in order to determine whether the correlation was related to the intrinsic status of tumor and independent of the tumor microenvironment. A significant positive correlation between PD-L1 and MET was also found in several cancer cell lines, including NSCLC, breast cancer, and sarcoma (Supplementary Figure S7), but not in others, such as stomach cancer and lung small cell carcinoma. The findings from the TCGA and CCLE analyses indicated that the positive correlation of PD-L1 and MET depends on the cellular origin of the tumor and the tumor microenvironment.

### 2.5. Landscape of MET, PD-L1 and T-cell Effector Molecule Expression in Human Cancers

**Figure 6:**
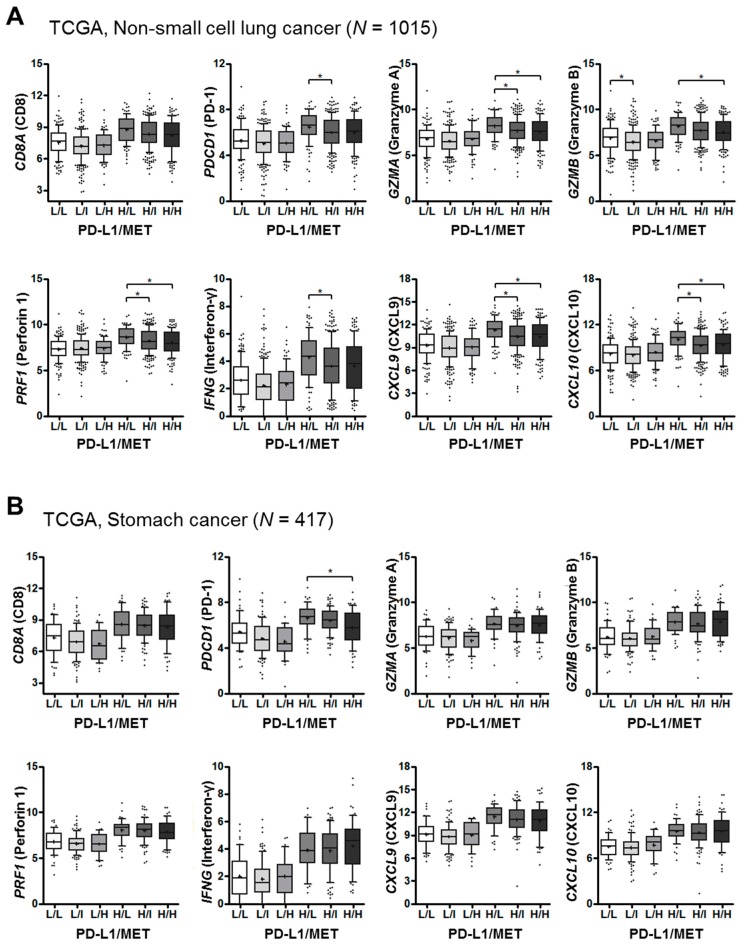
Expression of immune-related genes as function of PD-L1/MET expression in human cancer tissues (TCGA data analysis). The expression levels of T-cell effector molecules, including *CD8A, PDCD1* (PD-1), *GZMA* (granzyme A), *GZMB* (granzyme B), *PRF1* (perforin 1), *IFNG* (interferon-γ), *CXCL9*, and *CXCL10*, were comparatively analyzed according to the *CD274* (PD-L1) and *MET* expression status of the non-small cell lung (*n* = 1015) and gastric (*n* = 417) cancers listed in the TCGA dataset. The cancers were dichotomized into *CD274* (PD-L1) low and high groups, based on the median value, and trichotomized into *MET* low (<25th percentile), intermediate (25th–75th percentile), and high (>75th percentile) groups. The mRNA levels of T-cell effector molecules were compared by Kruskal–Wallis test and one-way ANOVA. The whiskers ranged from the 10th and to the 90th percentile. The middle line of the box is plotted at the median and “+” denotes the mean. Points below and above the whiskers are drawn as individual points. (Abbreviations: L, low; I, intermediate; H, high.). * *p* < 0.05.

The TCGA dataset was used to explore the landscape of MET, PD-1 ligand, and T-cell effector molecule expression in human cancers, specifically, the potential biological and clinical relevance of MET and PD-L1 in the tumor immune microenvironment of NSCLC and gastric cancer. In NSCLC, the expression of T-cell effector molecules, including PD-1 (*PDCD1*), granzyme A (*GZMA*), granzyme B (*GZMB*), perforin (*PRF1*), IFNγ (*IFNG*), *CXCL9*, and *CXCL10*, correlated inversely with MET expression, especially in the PD-L1^high^ vs. PD-L1^low^ group (Figure 6A). By contrast, the correlation between MET/PD-L1 and T-cell effector gene expression in gastric cancer was not significant (Figure 6B). These data suggest that MET overexpression is related to the poor anti-tumor immune response of PD-L1 expressing lung cancers.

## 3. Discussion

MET-mediated immune regulation in tumors was comprehensively investigated in a microarray analysis performed after the functional and genetic suppression of MET in Hs746T cells. In the analyzed tumor cells, the expression of genes involved in the immune response was significantly changed, as was that of genes possibly regulated by MET. Of note, the levels of co-inhibitory molecules, including PD-L1, PD-L2, B7-H3, and PVRL1, were decreased, and those of co-stimulatory molecules including 4-1BBL, OX40L, CD70, and ICAM1, increased by MET suppression via siRNA knockdown or functional inhibition. The changes in PD-L1, 4-1BBL, OX40L, and CD70 expression after MET activation and suppression were validated by qRT-PCR and western blot or flow cytometry analysis using three cell lines differing in their *MET* status and a human bronchial epithelial cell line. It was recently reported that the MET pathway is involved in PD-L1 up-regulation in cancer cells [20], but its role in the down-regulation of co-stimulatory molecules was not addressed. Our findings indicate a role for MET in the immune escape of tumors, via its ability to up-regulate co-inhibitory pathways and down-regulate co-stimulatory pathways.

Microarray analysis revealed that, in response to MET suppression, PD-L1 and PD-L2 were the most strongly down-regulated co-inhibitory molecules. MET and PD-L1 levels correlated positively in lung cancer, gastric cancer, and renal cell carcinoma as determined by IHC [17,18,21,22]. PD-L1 overexpression was observed in NSCLC with *MET* amplification and acquired resistance to EGFR TKI, both in this and in other studies [23,24]. However, the role of MET in PD-L1 expression in different tumor types is unclear [20,25,26]. In their study of *MET*-altered lung cancer and gastrointestinal cancer cell lines, Saigi et al. reported that MET activation induced PD-L1 expression independently of the IFNγ-JAK2 pathway [20]. Martin et al. also reported the involvement of MET in IFNγ-JAK2/STAT1-medaited PD-L1 induction and that MET inhibition hampered this pathway [25]. By contrast, Li et al. demonstrated that MET decreases PD-L1 expression in hepatocellular carcinoma by the GSK3β-mediated post-translational modification/degradation of PD-L1 protein, rather than by its transcriptional regulation [26]. In this study, MET was shown to transcriptionally up-regulate PD-L1 and its subsequent protein and surface expression, suggestive of a functional enhancement of PD-L1 by MET. However, because PD-L1 expression is regulated by cancer-cell-intrinsic (i.e., PD-L1 gene status and driver oncogenic pathways) and extrinsic (i.e., cytokine milieu) factors [27,28,29], the role of MET in PD-L1 induction might differ according to the tumor type and the tumor microenvironment. This possibility was supported by the results of the TCGA analysis (at the mRNA levels), which showed frequent but variable positive correlations between MET and PD-L1 expression in diverse human cancer types. These included lung and stomach cancers, as demonstrated in this study, and in previous studies using cell lines derived from these tumors [20,25], as well as bladder, breast and kidney cancers, but not in esophageal, colon, liver, mesothelial, lymphoid, and soft-tissue cancers. In lung adenocarcinoma, the genetic background seems to affect the relationship between MET and PD-L1; thus, in tumors with *KRAS* mutation or those lacking *EGFR* mutation/*KRAS* mutation/*ALK* translocation (so called triple-negative), but not *EGFR*-mutated tumors, there was a strong positive correlation between MET and PD-L1.

In this study, basal expression levels of PD-L1 in cell lines carrying *MET* alterations were variable and highest in Hs746T cells, harboring both *MET* mutation and amplification. In H1993 cells harboring only *MET* amplification, p-MET levels were comparable to those of Hs746T cells (Supplementary Figure S1), but PD-L1 expression was barely detectable on the same blot showing high levels in Hs746T cells. Thus, genetic alterations other than MET may also be involved in regulation of PD-L1 expression in *MET*-altered tumors.

Although a positive and causal relationship between MET and PD-L1 expression has been reported for in some cancer types [17,18,20,21,22], little is known about the functional role of MET in tumor immune evasion. While MET overexpression was associated with an unfavorable clinical outcome during EGFR-targeting therapy, it tended to be associated with more favorable clinical outcome in patients treated with PD-1/PD-L1-targeted therapy [4]. However, the influence of MET overexpression by the tumor on the efficacy of PD-1/PD-L1 blockade is unclear. Using an in vitro co-culture system, we showed that MET^high^/PD-L1^high^ Hs746T (gastric cancer) cells suppressed immune cell function, which was partially recovered by anti-PD-L1 blocking antibody and the clinically administered MET inhibitor crizotinib. Consistent with this result, TCGA analysis revealed an inverse correlation between MET overexpression and T-cell effector gene expression in PD-L1^high^ NSCLC, which suggested an immunosuppressive role of MET in the microenvironment of this tumor. This correlation was not observed in patients with gastric cancer, indicating a biologically different role for, or significance of, MET in immune evasion across different tumor types. Clinical trials for MET-targeted therapies are ongoing for patients with lung cancer and colorectal cancer based on the pro-tumorigenic role of MET (NCT 01982955, NCT 02132598 and NCT 02510001). Thus, MET may be an effective therapeutic target in combination therapy with PD-1/PD-L1 blockade, based on its pro-tumorigenic and immune suppressive role in cancer. The utility of MET-targeted therapy in combination with immune targets within the PD-1/PD-L1 pathway or other immune checkpoint molecules remained to be determined in preclinical and clinical studies.

In summary, this study demonstrated that MET has a role in tumor immune evasion via up-regulating the expression of co-inhibitory molecules, particularly PD-L1, and down-regulating the expression of co-stimulatory molecules. In vitro, MET overexpression in tumor cells contributed to the suppression of immune cell function, which was partially restored by treatment with a MET inhibitor or PD-L1 blockade. Our results also showed that MET overexpression was related to immunosuppressive features in the tumor microenvironment of PD-L1^high^ lung cancer. Combined MET-targeted therapy and immunotherapy should therefore be considered for patients with MET-dependent cancer. However, the therapeutic strategies likely to be most effective in a combined approach and the patients most likely to benefit from such approach must still be clarified.

## 4. Materials and Methods

### 4.1. Cell Lines and Reagents

The lung adenocarcinoma cell lines H596 (harboring the *MET* exon 14 skipping mutation) and H1993 (*MET* amplification), the gastric carcinoma cell line Hs746T (both the *MET* exon 14 skipping mutation and amplification), and the non-neoplastic human bronchial epithelial cell line BEAS-2B were used in this study. H596 and Hs746T cells were kindly provided by James G. Christensen (Mirati Therapeutics, San Diego, CA, USA), and H1993 cells by Sukjoon Yoon (Sookmyung Women’s University, Seoul, Korea). BEAS-2B (ATCC CRL-9609) cells were purchased from the American Type Culture Collection (Manassas, VA, USA). Hs746T cells were maintained in DMEM supplemented with 10% fetal bovine serum (FBS) and 1% antibiotics, while H596 and H1993 cells were maintained in RPMI-1640 medium supplemented with 10% FBS and 1% antibiotics, and BEAS-2B cells were in DMEM/F12 (1:1) with 5% FBS and 1% antibiotics. All cell lines were kept in a humidified atmosphere of 5% CO_2_ at 37 °C. PHA665752 (a MET inhibitor) was purchased from Sigma-Aldrich (St. Louis, MO, USA), crizotinib came from Selleckchem (Houston, TX, USA), and rhHGF was from ProSpec-Tany TechnoGene Ltd. (Ness Ziona, Israel).

### 4.2. Small Interfering RNA (siRNA) Transfections

Three types of specific siRNAs targeting human MET and a scrambled (sc) siRNA were synthesized by Bioneer (Daejeon, Korea) (sequences in Supplementary Table S1). Hs746T and H1993 cells seeded in 6-well plates and grown to 60–70% confluency were transfected with siRNA using Lipofectamine 2000 (Invitrogen, Carlsbad, CA, USA) in an Opti-MEM (Qiagen, Germantown, MD, USA). After 6 h, the medium was replaced with complete medium and the cells were harvested 48 h after transfection.

### 4.3. Oligonucleotide Microarray Analysis

Hs746T cells were treated with MET inhibitor or transfected with MET siRNA, after which total RNA was extracted using the TRIzol reagent (Invitrogen). Microarray analysis and data acquisition were performed using an Agilent human 44K (V2) gene expression microarray by E-BIOGEN (Seoul, Korea) according to the manufacturer’s instructions. Briefly, target cRNA probes and hybridization were synthesized using the LowInput QuickAmp labeling kit (Agilent Technologies, Santa Clara, CA, USA). After the labeling efficiency was confirmed, 1650 ng of cyanine 3-labeled cRNA target was subject to fragmentation by the addition of 10× blocking agent and 25× fragmentation buffer. The fragmented cRNA was resuspended in 2× hybridization buffer and used directly in the assembled Agilent human 44K (V2) gene expression microarray. The arrays were hybridized at 65 °C for 17 h in an Agilent hybridization oven and then washed. The hybridization images were captured and quantitated using an Agilent DNA microarray scanner and Agilent feature extraction software 10.7. The average fluorescence intensity for each spot was calculated after subtracting the local background intensity. All data were normalized and fold-changed genes were selected using GeneSpringGX 7.3.1 (Agilent Technologies, Santa Clara, CA, USA). Functional annotation of the genes was performed according to the Gene Ontology Consortium (http://www.geneontology.org/index.shtml) by GeneSpringGX 7.3.1.

### 4.4. Quantitative Real-Time Reverse Transcription PCR (qRT-PCR)

Total RNA (1 μg) was reverse-transcribed using a cDNA synthesis kit (Takara Bio, Otsu, Japan). The qRT-PCR was performed using SYBR green (Takara Bio, Otsu, Japan), primers (Supplementary Table S1), and a Step One Plus thermal cycler (Applied Biosystems, Foster City, CA, USA). Relative mRNA expression was measured as the fold-change using the 2^−∆∆*C*t^ method, in which ∆∆Ct = (Ct of molecule, treated − Ct of GAPDH, treated) − (Ct of molecule, control − Ct of GAPDH, control).

### 4.5. Flow Cytometry

Cells were stained with the phycoerythrin (PE)-conjugated anti-PD-L1 antibody (eBioscience, San Diego, CA, USA), PE-conjugated anti-CD70 antibody (Invitrogen, Carlsbad, CA, USA), PE-conjugated anti-OX40L antibody (Biolegend, San Diego, CA, USA), and APC-conjugated anti-4-1BBL antibody (Biolegend) or the isotype control (mouse IgG1κ, eBioscience) and subjected to flow cytometry using a FACS Canto (BD Biosciences, Franklin Lakes, NJ, USA).

### 4.6. Western Blot

Total cellular protein was extracted using RIPA lysis buffer supplemented with phosphatase and protease inhibitor cocktail and EDTA (Sigma-Aldrich, St. Louis, MO, USA). The protein samples (20–40 µg each) were subjected to SDS-PAGE and transferred to a PVDF membrane (Millipore, Bedford, MA, USA). Immunoblotting was performed using antibodies against MET (#4250S, Cell Signaling Technology, Danvers, MA, USA; diluted at 1:1000 in 5% skim milk), phospho-MET (p-MET) (Tyr1234/1235) (#3126S, Cell Signaling Technology, Danvers, MA, USA; 1:500 in 5% bovine serum albumin), PD-L1 (#1368S, clone E1L3N, Cell Signaling Technology, Danvers, MA, USA; 1:1000 in 5% skim milk), PD-L2 (#MAB1224-100, clone 176611, R&D systems, Minneapolis, MN, USA; 1:500 in 5% skim milk), or β-actin (#sc-47778, Santa Cruz Biotechnology, Santa Cruz, CA, USA; 1:5000 in 5% skim milk). The bound antibody was visualized using a chemiluminescence kit (Amersham Pharmacia Biotech, Uppsala, Sweden).

### 4.7. Co-Culture of Human Peripheral Blood Mononuclear Cells (Pbmcs) and Tumor Cells

Human PBMCs obtained from the heparinized peripheral blood of healthy volunteers were isolated using Ficoll-Paque Plus (GE Healthcare, Uppsala, Sweden) density gradient separation. The cells were activated for 8 h with 50 ng phorbol 12-myristate 13-acetate (PMA)/mL and 1 µg ionomycin/mL. Surface expression of PD-1 on T cells was determined by flow cytometry using PE-conjugated anti-human PD-1 (CD279) antibody (eBioscience, San Diego, CA, USA). Hs746T tumor cells were seeded in 6-well plates and cultured in RPMI-1640 medium before their co-culture with activated PBMCs. Alternatively, Hs746T cells were pretreated for 24 h with crizotinib or anti-PD-L1 blocking antibody. PBMCs and Hs746T cells were co-cultured at a ratio of 2:1 and for 16 h, after which the PMBCs and culture medium were harvested for qRT-PCR and ELISA for IFNγ, respectively. This study was performed in accordance with the World Medical Association Declaration of Helsinki and was approved by the Institutional Review Board (IRB) of Seoul National University Hospital (SNUH) (H-1408-007-598).

### 4.8. Enzyme-Linked Immunosorbent Assay (ELISA) for IFNγ

IFNγ levels were measured in the cell-free supernatants from the co-culture system using a human IFNγ ELISA kit (R&D systems, Minneapolis, MN, USA, #DY285-05) according to the manufacturer’s protocol.

### 4.9. Immunohistochemistry (IHC)

Tissue microarrays (2-mm-diameter cores) were constructed from the formalin-fixed paraffin-embedded tumor tissues of 789 patients with NSCLC who underwent surgical resection of the tumor and were then followed-up at SNUH. IHC was performed using rabbit anti-PD-L1 (clone E1L3N) and anti-MET (clone SP44, Ventana Medical Systems, Tucson, AZ, USA), and mouse anti-PD-L2 (clone 176611, R&D systems, Minneapolis, MN, USA) antibodies together with the Ventana Benchmark XT autostainer. MET IHC was scored by the criteria used in the clinical trials of a MET inhibitor as follows: Zero (0), absence of staining or any staining in <50% of the tumor cells; 1, weak to moderate intensity staining in ≥50% of the tumor cells; 2, moderate to strong intensity staining in ≥50% of the tumor cells; 3, strong intensity staining in ≥50% of the tumor cells. PD-L1 IHC was evaluated based on the intensity and proportion of membranous and/or cytoplasmic staining in tumor cells, and scored as follows: Zero (0), negative; 1, weak or moderate in <10% of tumor cells; 2, moderate in ≥10% of tumor cells; 3, strong in ≥10% of tumor cells. The clinicopathological features of the patients are summarized in Supplementary Table S2. Pathologic tumor-node-metastasis (TNM) staging was based on the 7th American Joint Committee on Cancer. *EGFR* and *KRAS* mutations were examined using direct sequencing or peptide nucleic acid-clamping PCR. *ALK* translocation and *MET* gene copy number were evaluated using fluorescence in situ hybridization (FISH) as previously reported [17]. This study was performed in accordance with the World Medical Association Declaration of Helsinki and was approved by the IRB of SNUH (H-1404-100-572). Informed consent for participation was waived by the IRB of SNUH given the retrospective design of the study and the use of archived material.

### 4.10. Cancer Cell Line Encyclopedia (CCLE) and the Cancer Genome Atlas (TCGA) Data Analyses

The relationship between MET and PD-L1 expression in human cancer cell lines was explored in a CCLE analysis (https://portals.broadinstitute.org/ccle). Level 3 data of the TCGA, downloaded from the UCSC Cancer Browser (https://genome-cancer.ucsc.edu) on 3 June 2015, were used. TCGA data included clinical information and mRNA expression data obtained by RNAseq (Illumina HiSeq V2 platform, San Diego, CA, USA). Samples of 14 cancer types (*n* = 5470) were included in our analysis of the association between MET and PD-L1. Data from 1015 patients with NSCLC were analyzed to explore the expression pattern of immune-related molecules according to the expression status of PD-L1 and MET.

### 4.11. Statistical Analysis

All statistical analyses were performed using SPSS software (version 21; IBM Corp., New York, NY, USA). For patient sample data and in vitro studies, the expression levels of several continuous variables, including MET and PD-L1, were compared using Student *t*-tests. For CCLE and TCGA data, the statistical significance was calculated using Spearman’s correlation or Pearson’s correlation analyses. The mRNA levels of T-cell effector response-related genes according to *CD274* and MET status were compared using Kruskal–Wallis tests and one-way ANOVA. Statistical significance in the difference of categorical values was analyzed using Pearson’s chi-squared tests. Two-sided *p* values <0.05 were considered statistically significant.

## Figures and Tables

**Table 1:** Correlations between MET and PD-1 ligand expression in human cancer tissues (TCGA data analysis).

Tumor Type (*n*)	MET	PD-L1	r *^,1^	*p* ^1^	PD-L2	r *^,2^	*p* ^2^
	Mean (Median)	Mean (Median)			Mean (Median)		
HNSCC (*n* = 519)	11.05 (11.12)	6.36 (6.35)	0.11	0.012	6.65 (6.63)	0.174	0.000
NSCLC (*n* = 1015)	11.40 (11.45)	6.24 (6.07)	0.219 †	0.000	6.50 (6.54)	0.181	0.000
Lung ADC (*n* = 513)	11.66 (11.84)	6.05 (5.98)	0.401	0.000	6.46 (6.50)	0.266	0.000
Lung SqCC (*n* = 502)	11.13 (11.24)	6.43 (6.22)	0.144	0.001	6.53 (6.57)	0.085	0.058
Eso cancer (*n* = 184)	11.53 (11.47)	5.43 (5.35)	0.02	0.792	5.46 (5.41)	−0.046	0.535
Stomach cancer (*n* = 415)	11.31 (11.25)	5.35 (5.30)	0.137	0.005	5.84 (6.00)	0.015	0.754
Colon ADC (*n* = 380)	11.77 (11.74)	4.28 (4.14)	−0.098	0.056	4.44 (4.56)	−0.169	0.001
HCC (*n* = 371)	11.57 (11.62)	3.22 (3.20)	−0.085	0.102	4.37 (4.28)	−0.107	0.039
Breast cancer (*n* = 1095)	8.31 (8.43)	4.43 (4.38)	0.238	0.000	5.96 (6.07)	0.383	0.000
Kidney RCC (*n* = 534)	12.60 (12.59)	5.35 (5.38)	0.19	0.000	6.40 (6.45)	0.108	0.013
Bladder cancer (*n* = 407)	10.56 (10.59)	4.69 (4.32)	0.385	0.000	4.96 (4.99)	0.355	0.000
GBM (*n* = 157)	7.43 (7.40)	4.88 (4.88)	0.226	0.004	6.63 (6.81)	0.237	0.003
DLBCL (*n* = 48)	7.03 (6.83)	6.30 (6.05)	0.12	0.415	8.25 (8.19)	−0.045	0.762
Mesothelioma (*n* = 87)	11.70 (12.13)	4.76 (4.39)	0.022	0.842	6.70 (6.55)	−0.079	0.468
Sarcoma (*n* = 258)	8.47 (8.23)	4.11 (4.06)	0.124	0.047	7.06 (7.29)	0.122	0.05

Abbreviations: ADC, adenocarcinoma; DLBCL, diffuse large B-cell lymphoma; Eso, esophageal; GBM, glioblastoma; HCC, hepatocellular carcinoma; HNSCC, head and neck squamous cell carcinoma; NSCLC, non-small cell lung cancer; RCC, renal cell carcinoma; SqCC, squamous cell carcinoma. ^1^, correlations between MET and PD-L1; ^2^, correlations between MET and PD-L2; *, Pearson’s r. †, Spearman’s rho.

## Supplementary Materials

Supplementary materials can be found at https://www.mdpi.com/1422-0067/20/17/4287/s1.

## Abbreviations

CCLE	Cancer Cell Line Encyclopedia
IFNγ	interferon-γ
IHC	immunohistochemistry
NSCLC	non-small cell lung cancer
PBMC	peripheral blood mononuclear cells
PD-1	programmed cell death-1
PD-L1	programmed cell death-1-ligand 1
PD-L2	programmed cell death-1-ligand 2
PMA	phorbol 12-myristate 13-acetate
qRT-PCR	quantitative reverse transcriptase–polymerase chain reaction
rhHGF	recombinant human hepatocyte growth factor
TCGA	The Cancer Genome Atlas
TKI	tyrosine kinase inhibitor

## References

[1] Gherardi E., Birchmeier W., Birchmeier C., Vande Woude G. (2012). Targeting MET in cancer: Rationale and progress. Nat. Rev. Cancer.

[2] Gelsomino F., Facchinetti F., Haspinger E.R., Garassino M.C., Trusolino L., De Braud F., Tiseo M. (2014). Targeting the MET gene for the treatment of non-small-cell lung cancer. Crit. Rev. Oncol. Hematol..

[3] Westover D., Zugazagoitia J., Cho B.C., Lovly C.M., Paz-Ares L. (2018). Mechanisms of acquired resistance to first- and second-generation EGFR tyrosine kinase inhibitors. Ann. Oncol..

[4] Reis H., Metzenmacher M., Goetz M., Savvidou N., Darwiche K., Aigner C., Herold T., Eberhardt W.E., Skiba C., Hense J. (2018). MET Expression in Advanced Non-Small-Cell Lung Cancer: Effect on Clinical Outcomes of Chemotherapy, Targeted Therapy, and Immunotherapy. Clin. Lung Cancer.

[5] Schildhaus H.U., Schultheis A.M., Ruschoff J., Binot E., Merkelbach-Bruse S., Fassunke J., Schulte W., Ko Y.D., Schlesinger A., Bos M. (2015). MET amplification status in therapy-naive adeno- and squamous cell carcinomas of the lung. Clin. Cancer Res..

[6] Kong-Beltran M., Seshagiri S., Zha J., Zhu W., Bhawe K., Mendoza N., Holcomb T., Pujara K., Stinson J., Fu L. (2006). Somatic mutations lead to an oncogenic deletion of met in lung cancer. Cancer Res..

[7] Onozato R., Kosaka T., Kuwano H., Sekido Y., Yatabe Y., Mitsudomi T. (2009). Activation of MET by gene amplification or by splice mutations deleting the juxtamembrane domain in primary resected lung cancers. J. Thorac. Oncol. Off. Publ. Int. Assoc. Study Lung Cancer.

[8] Awad M.M., Oxnard G.R., Jackman D.M., Savukoski D.O., Hall D., Shivdasani P., Heng J.C., Dahlberg S.E., Janne P.A., Verma S. (2016). MET Exon 14 Mutations in Non-Small-Cell Lung Cancer Are Associated With Advanced Age and Stage-Dependent MET Genomic Amplification and c-Met Overexpression. J. Clin. Oncol..

[9] Liu X., Jia Y., Stoopler M.B., Shen Y., Cheng H., Chen J., Mansukhani M., Koul S., Halmos B., Borczuk A.C. (2016). Next-Generation Sequencing of Pulmonary Sarcomatoid Carcinoma Reveals High Frequency of Actionable MET Gene Mutations. J. Clin. Oncol..

[10] Kwon D., Koh J., Kim S., Go H., Kim Y.A., Keam B., Kim T.M., Kim D.W., Jeon Y.K., Chung D.H. (2017). MET exon 14 skipping mutation in triple-negative pulmonary adenocarcinomas and pleomorphic carcinomas: An analysis of intratumoral MET status heterogeneity and clinicopathological characteristics. Lung Cancer.

[11] Tong J.H., Yeung S.F., Chan A.W., Chung L.Y., Chau S.L., Lung R.W., Tong C.Y., Chow C., Tin E.K., Yu Y.H. (2016). MET amplification and exon 14 splice site mutation define unique molecular subgroups of Non-small Cell Lung Carcinoma with poor prognosis. Clin. Cancer Res..

[12] Pardoll D.M. (2012). The blockade of immune checkpoints in cancer immunotherapy. Nat. Rev. Cancer.

[13] Zou W., Wolchok J.D., Chen L. (2016). PD-L1 (B7-H1) and PD-1 pathway blockade for cancer therapy: Mechanisms, response biomarkers, and combinations. Sci. Transl. Med..

[14] Darvin P., Toor S.M., Sasidharan Nair V., Elkord E. (2018). Immune checkpoint inhibitors: Recent progress and potential biomarkers. Exp. Mol. Med..

[15] Prelaj A., Tay R., Ferrara R., Chaput N., Besse B., Califano R. (2019). Predictive biomarkers of response for immune checkpoint inhibitors in non-small-cell lung cancer. Eur. J. Cancer.

[16] Fumet J.D., Richard C., Ledys F., Klopfenstein Q., Joubert P., Routy B., Truntzer C., Gagne A., Hamel M.A., Guimaraes C.F. (2018). Prognostic and predictive role of CD8 and PD-L1 determination in lung tumor tissue of patients under anti-PD-1 therapy. Br. J. Cancer.

[17] Koh J., Go H., Keam B., Kim M.Y., Nam S.J., Kim T.M., Lee S.H., Min H.S., Kim Y.T., Kim D.W. (2015). Clinicopathologic analysis of programmed cell death-1 and programmed cell death-ligand 1 and 2 expressions in pulmonary adenocarcinoma: Comparison with histology and driver oncogenic alteration status. Mod. Pathol..

[18] Kim M.Y., Koh J., Kim S., Go H., Jeon Y.K., Chung D.H. (2015). Clinicopathological analysis of PD-L1 and PD-L2 expression in pulmonary squamous cell carcinoma: Comparison with tumor-infiltrating T cells and the status of oncogenic drivers. Lung Cancer.

[19] Maroun C.R., Rowlands T. (2014). The Met receptor tyrosine kinase: A key player in oncogenesis and drug resistance. Pharmacol. Ther..

[20] Saigi M., Alburquerque-Bejar J.J., Mc L.-F.A., Pereira C., Pros E., Romero O.A., Baixeras N., Esteve-Codina A., Nadal E., Brambilla E. (2018). MET-oncogenic and JAK2-inactivating alterations are independent factors that affect regulation of PD-L1 expression in lung cancer. Clin. Cancer Res..

[21] Shin S.J., Jeon Y.K., Kim P.J., Cho Y.M., Koh J., Chung D.H., Go H. (2016). Clinicopathologic Analysis of PD-L1 and PD-L2 Expression in Renal Cell Carcinoma: Association with Oncogenic Proteins Status. Ann. Surg. Oncol..

[22] Xing X., Guo J., Wen X., Ding G., Li B., Dong B., Feng Q., Li S., Zhang J., Cheng X. (2018). Analysis of PD1, PDL1, PDL2 expression and T cells infiltration in 1014 gastric cancer patients. Oncoimmunology.

[23] Han J.J., Kim D.W., Koh J., Keam B., Kim T.M., Jeon Y.K., Lee S.H., Chung D.H., Heo D.S. (2016). Change in PD-L1 Expression After Acquiring Resistance to Gefitinib in EGFR-Mutant Non-Small-Cell Lung Cancer. Clin. Lung Cancer.

[24] Demuth C., Andersen M.N., Jakobsen K.R., Madsen A.T., Sorensen B.S. (2017). Increased PD-L1 expression in erlotinib-resistant NSCLC cells with MET gene amplification is reversed upon MET-TKI treatment. Oncotarget.

[25] Martin V., Chiriaco C., Modica C., Acquadro A., Cortese M., Galimi F., Perera T., Gammaitoni L., Aglietta M., Comoglio P.M. (2019). Met inhibition revokes IFNgamma-induction of PD-1 ligands in MET-amplified tumours. Br. J. Cancer.

[26] Li H., Li C.W., Li X., Ding Q., Guo L., Liu S., Liu C., Lai C.C., Hsu J.M., Dong Q. (2019). MET Inhibitors Promote Liver Tumor Evasion of the Immune Response by Stabilizing PDL1. Gastroenterology.

[27] Topalian S.L., Drake C.G., Pardoll D.M. (2015). Immune checkpoint blockade: A common denominator approach to cancer therapy. Cancer Cell.

[28] Akbay E.A., Koyama S., Carretero J., Altabef A., Tchaicha J.H., Christensen C.L., Mikse O.R., Cherniack A.D., Beauchamp E.M., Pugh T.J. (2013). Activation of the PD-1 pathway contributes to immune escape in EGFR-driven lung tumors. Cancer Discov..

[29] Koh J., Jang J.Y., Keam B., Kim S., Kim M.Y., Go H., Kim T.M., Kim D.W., Kim C.W., Jeon Y.K. (2016). EML4-ALK enhances programmed cell death-ligand 1 expression in pulmonary adenocarcinoma via hypoxia-inducible factor (HIF)-1alpha and STAT3. Oncoimmunology.

