# Supplementary material for: MET Receptor Tyrosine Kinase Regulates the Expression of Co-Stimulatory and Co-Inhibitory Molecules in Tumor Cells and Contributes to PD-L1-Mediated Suppression of Immune Cell Function
Source: Int J Mol Sci. 2019 Sep 1;20(17):4287. doi: 10.3390/ijms20174287 (PMC6747314; doi:10.3390/ijms20174287)
Supplement: Supplementary file 1 [file ijms-20-04287-s001.zip › ijms-566225 suppl for final/ijms-566225 suppl for final.pdf]

Supplementary Tables

Supplementary Table S1. Sequences of Small Interfering RNAs for Transfection and Primers Used for qRT-PCR.

**Small Interfering RNAs (siRNAs)**			
MET	siRNA-1	sense	5′-GAGCCAGCCUGAAUGAUGA-3′
		antisense	5′-UCAUCAUUCAGGCUGGCUC-3′
	siRNA-2	sense	5′- GAACAGCGAGCUAAAUAUA -3′
		antisense	5′- UAUAUUUAGCUCGCUGUUC -3′
	siRNA-3	sense	5′-GAACUGGUGUCCCGGAUAU-3′
		antisense	5′- AUAUCCGGGACACCAGUUC -3′
Scrambled siRNA		sense	5′-GGAGCAACGAGGAUUACCU-3′
		antisense	5′-AGGUAAUCCUCGUUGCUCC -3′

**Primers for qRT-PCR**		
MET	Forward	5′-TGATGATGAGGTGGACACA-3′
	Reverse	5′-CTATGGCAAGGAGCAAAGA-3′
CD274 (PD-L1)	Forward	5′-TATGGTGGTGGTGCCGACTACAA-3′
	Reverse	5′-TGGCTCCCAGAATTACCAAG-3′
TNFSF9 (4-1BBL)	Forward	CCTACATCTGCCTGCACTTCTC
	Reverse	TGATGACTGAGTTGTTCTGCACC
TNFSF4 (OX-40L)	Forward	GGCGTCCATCTTCACACTGA
	Reverse	CACCCAGGCTGGACGTTATT
CD70	Forward	GTCACTTGGGTGGGACGTAG
	Reverse	GATGGATACGTAGCTGCCCC
IFNG (IFNγ)	Forward	5′-CTAATTATTCGGTAACTGACTTGA-3′
	Reverse	5′-ACAGTTCAGCCATCACTTGGA-3′
GAPDH	Forward	5′-CCCTTCATTGACCTACCTCAACTACAT-3′
	Reverse	5′-ACGATACCAAAGTTGTCATGGAT-3′

Supplementary Table S2. Clinicopathological Parameters of Patients with NSCLC.

Clinicopathological Parameter	Adenocarcinoma (n = 789) (%)
Median age at surgery (range)	65 (23 -88)
Sex	
Male	567 (71.9)
Female	222 (28.1)
Smoking*	
Never	262 (34.0)
Ever	509 (66.0)
Histology	
Adenocarcinoma	387 (49.0)
Squamous cell carcinoma	385 (48.8)
Others	17 (2.2)
T stage*	
T1	270 (34.3)
T2	386 (49.0)
T3	88 (11.2)
T4	44 (5.5)
N stage*	
N0	499 (63.8)
N1-2	283 (36.2)
Stage*	
I	370 (47.3)
II	207 (26.5)
III	180 (23.0)
IV	25 (3.2)
EGFR mutation*	
No mutation	325 (63.6)
Mutation	186 (36.4)
KRAS mutation*	
No mutation	112 (90.3)
Mutation	12 (9.7)
ALK translocation*	
No translocation	511 (96.1)
Translocation	21 (3.9)
MET FISH*	
Others	226 (64.6)
High polysomy	99 (28.3)
Amplification	25 (7.1)
MET IHC	
0	516 (65.4)
1	154 (19.5)

2	89 (11.3)
3	30 (3.8)
PD-L1 IHC score	
0	139 (17.6)
1	304 (38.5)
2	274 (34.7)
3	72 (9.1)
PD-L2 IHC score	
0	141 (17.9)
1	309 (39.2)
2	262 (33.2)
3	77 (9.8)

* Some cases have missing values.

Supplementary Figure Legends

Supplementary Figure S1. Basal expression of MET, p-MET and PD-L1 in cancer cell lines. Basal expressions of MET, p-MET, and PD-L1 were evaluated using western blot in H596, Hs746T, and H1993 cells.

Supplementary Figure S2. Gene Ontology (GO) analysis of the genes up- or down-regulated by MET suppression in Hs746T cells. Hs746T cells were treated with a MET inhibitor PHA665752 or transfected with MET-specific siRNA1. **(A)** The efficacies of MET-specific siRNAs were determined by western blot. A sample transfected with MET-specific siRNA1 was submitted to microarray analysis. **(B)** GO analysis for the genes significantly changed more than 2-fold by PHA665752 treatment compared to DMSO treatment (as a control). **(C)** GO analysis for genes significantly changed more than 2-fold by MET siRNA transfection compared to sc siRNA transfection (as a control). The bar graphs represent the percentages of significantly up- (orange) or down-regulated (blue) genes in each gene set.

Supplementary Figure S3. Changes in The Expression of Co-Stimulatory and Co-Inhibitory Molecules in Human Non-Neoplastic Bronchial Epithelial Cells After rhHGF Treatment. (A) BEAS-2B cells maintained in serum-free medium for 6 h were treated with 100 ng rhHGF/mL for 12 h. The changes of *TNFSF9* (4-1BBL), *TNFSF4* (OX40L), *CD70,* and *CD274* (PD-L1) mRNA expression were then determined by qRT-PCR. **(B)** The surface expression of these molecules were analyzed by flow cytometry and mean fluorescence intensity (MFI) was plotted. **(C)** The changes in MET, p-MET, and PD-L1 total protein expression were examined by western blot. Data represent the mean ± SEM of at least three independent experiments **(A–B)**. All p values were calculated using unpaired Student's t-tests. * $p < 0.05$, ** $p < 0.01$.

Supplementary Figure S4. Changes in co-stimulatory and co-inhibitory molecules in human cancer cells after MET inhibitors treatment or MET-specific siRNA transfection. (A) Hs746T cells were treated with 400 nM PHA665752 (a MET inhibitor) for 24 h. Changes in total MET, p-MET, PD-L1, and PD-L2 protein expression were analyzed by western blot. **(B)** Hs746T cells were treated with crizotinib (a MET inhibitor) at the indicated concentrations for 24 h and the changes in *CD274* (PD-L1) mRNA expression were determined by qRT-PCR. **(C)** Hs746T cells were treated with 10 nM crizotinib for indicated times and the changes in PD-L1 protein expression were analyzed by western blot. **(D)** Changes in total MET expression in Hs746T cells transfected with MET-specific siRNAs were analyzed by western blot and quantified by densitometry. The relative mRNA levels of molecules are presented as the fold-change compared to the control ($2^{-\Delta\Delta Ct}$). Data are presented as the mean ± SEM of at least three independent experiments or are representative of three independent experiments. All p values were calculated using unpaired Student's t-tests. * $p < 0.05$, ** $p < 0.01$, *** $p < 0.001$.

Supplementary Figure S5. **Schematic procedure of co-culture experiment.** Procedure of co-culture experiment using peripheral blood mononuclear cells (PBMCs) and Hs746T cells is schematically depicted in upper panel. PBMCs were activated with 50 ng PMA/mL and 1 μg ionomycin/mL for 8 h. Surface expression of PD-1 on T cells was determined by flow cytometry ($CD3^+$ T cell gating by APC-conjugated anti-CD3 antibody) (lower).

Supplementary Figure S6. Co-culture of PBMCs and MET^{high}/PD-$L1^{high}$ Hs746T cells. PBMCs were stimulated by PMA and ionomycin, as in Supplementary Figure S5. Hs746T cells pre-treated for 24 h with 10 nM crizotinib or 40 μg anti-PD-L1 blocking antibody/mL were co-culture with stimulated PBMCs for 16 h. PBMCs were harvested and expression of IFNγ mRNA was analyzed by qRT-PCR. Relative levels of IFNγ mRNA are presented as the fold-change compared to the control ($2^{-\Delta\Delta Ct}$). Data

are presented as the mean ± SEM of at least three independent experiments. All p values were calculated using unpaired Student's *t*-tests. * $p < 0.05$, ** $p < 0.01$, *** $p < 0.001$.

Supplementary Figure S7. Correlations between MET and PD-L1 expression in human cancer cell lines from Cancer Cell Line Encyclopedia (CCLE). The correlation between MET and *CD274* (PD-L1) mRNA levels was analyzed in human cancer cell lines from Cancer Cell Line Encyclopedia (CCLE) according to the cancer types. The statistical significance was calculated using a Pearson's correlation analysis. Abbreviation: ADC, adenocarcinoma; CA, carcinoma; HN, head and neck; NSCLC, non-small cell lung cancer; RCC, renal cell carcinoma; SCC, small cell carcinoma; SQCC, squamous cell carcinoma.
