# Supplementary material for: MET Receptor Tyrosine Kinase Regulates the Expression of Co-Stimulatory and Co-Inhibitory Molecules in Tumor Cells and Contributes to PD-L1-Mediated Suppression of Immune Cell Function
Source: Int J Mol Sci. 2019 Sep 1;20(17):4287. doi: 10.3390/ijms20174287 (PMC6747314; doi:10.3390/ijms20174287)

Supplementary Figure S1

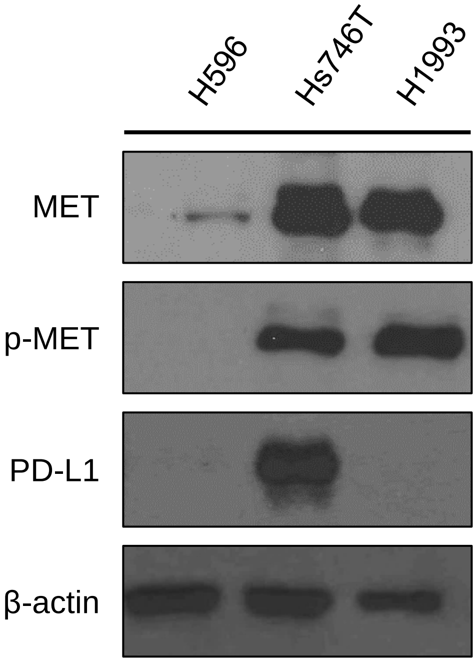

Supplementary Figure S2

A

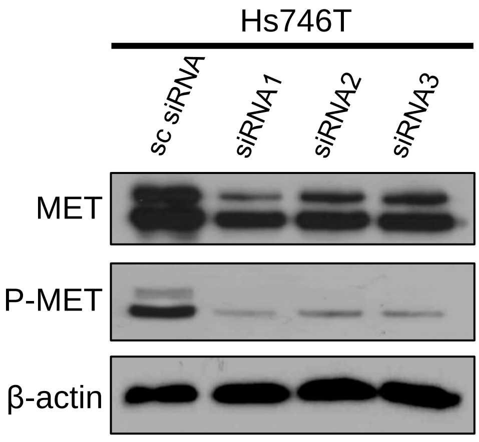

B Hs746T PHA665752 vs DMSO

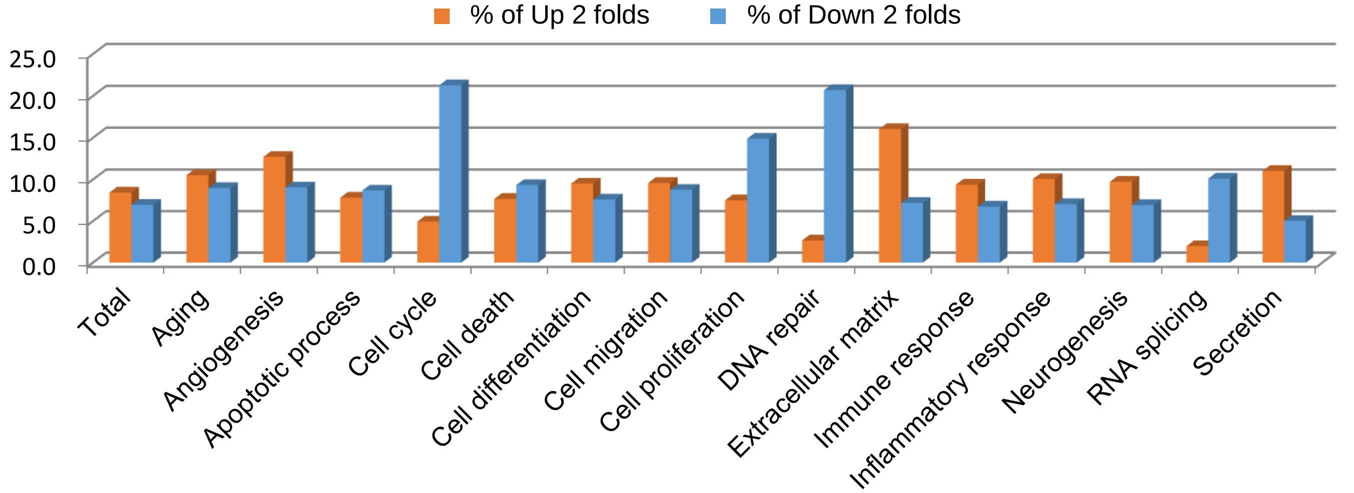

C Hs746T MET siRNA vs sc siRNA

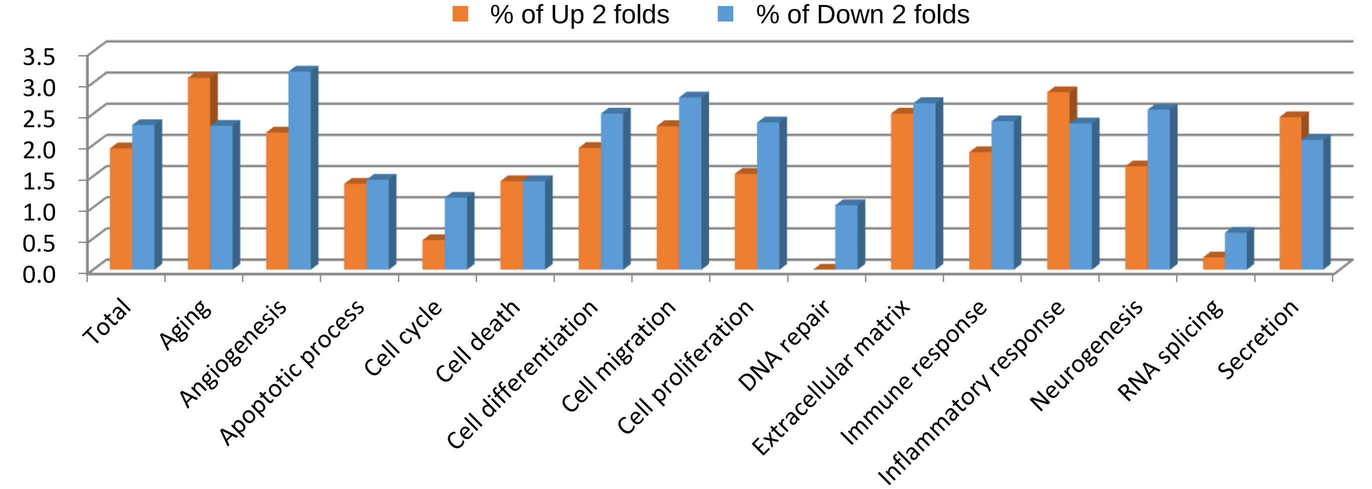

Supplementary Figure S3

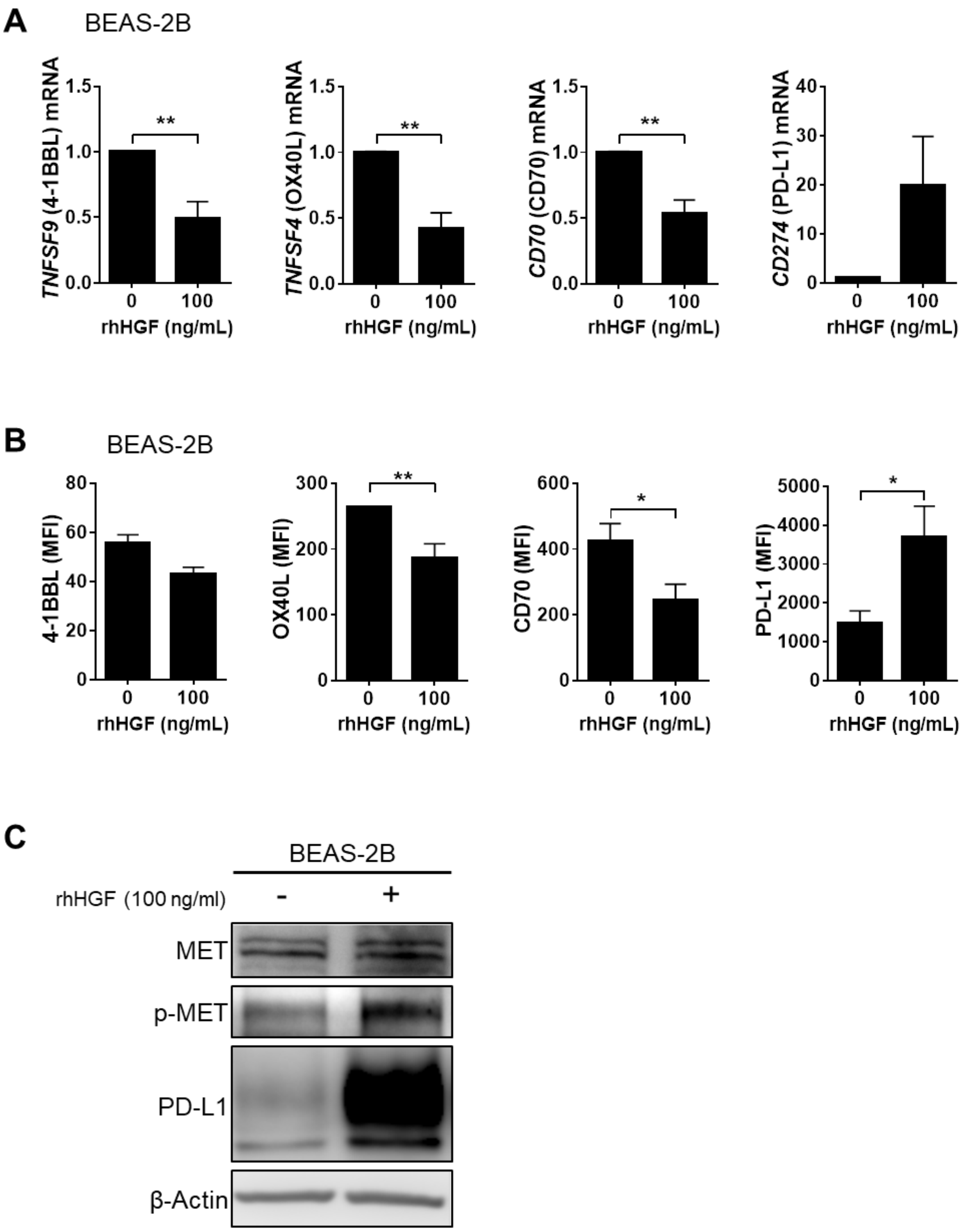

Supplementary Figure S4

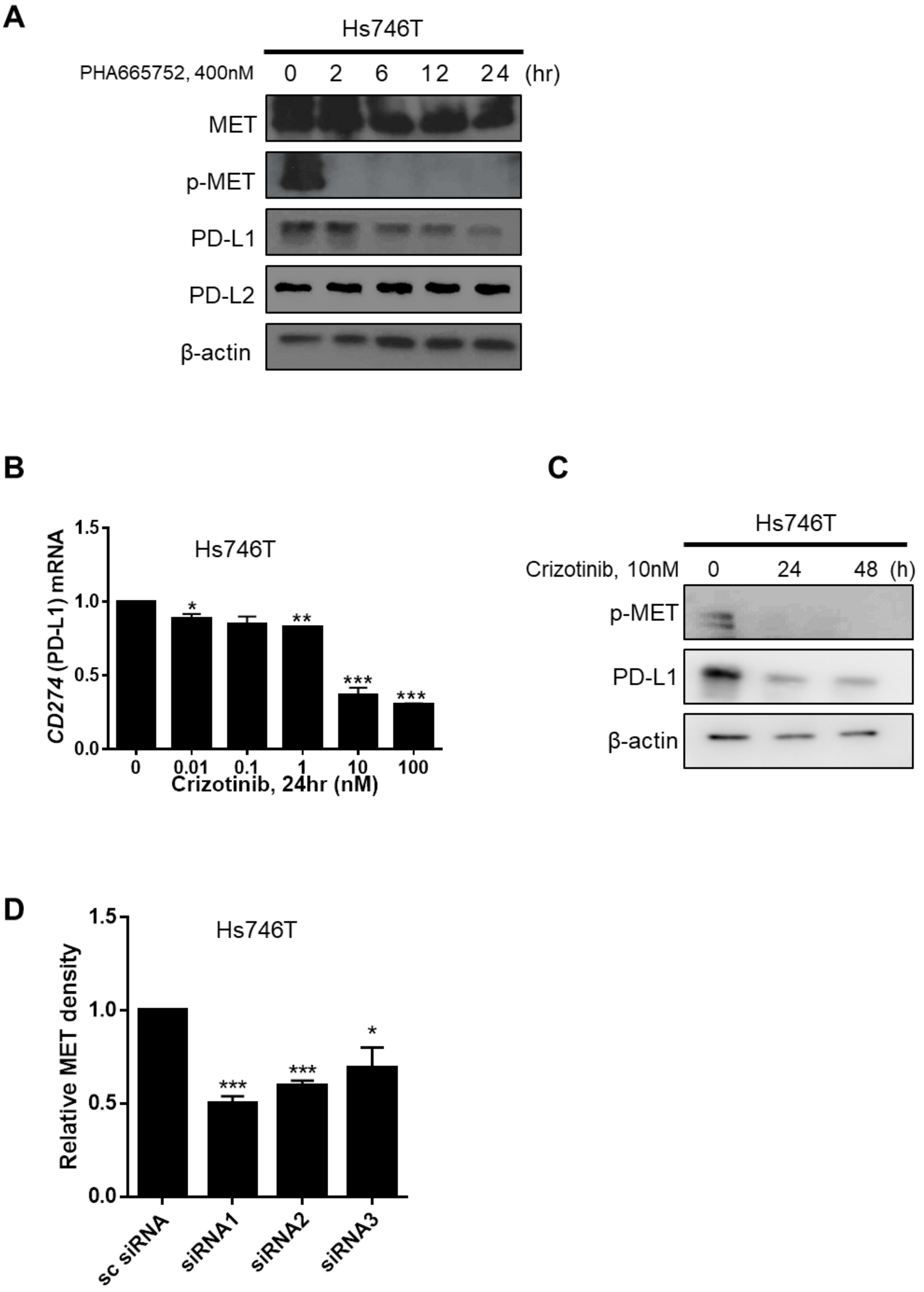

Supplementary Figure S5

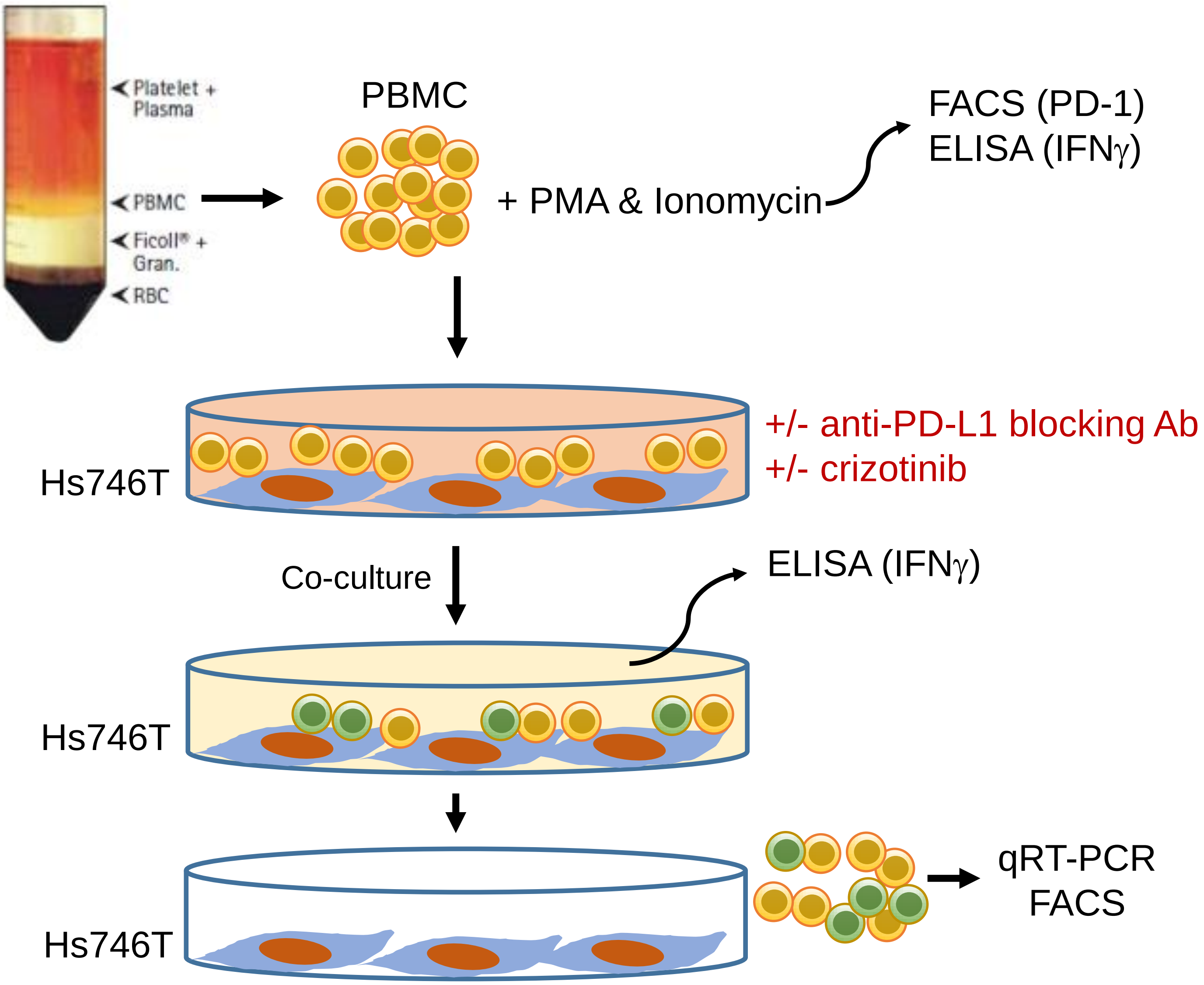

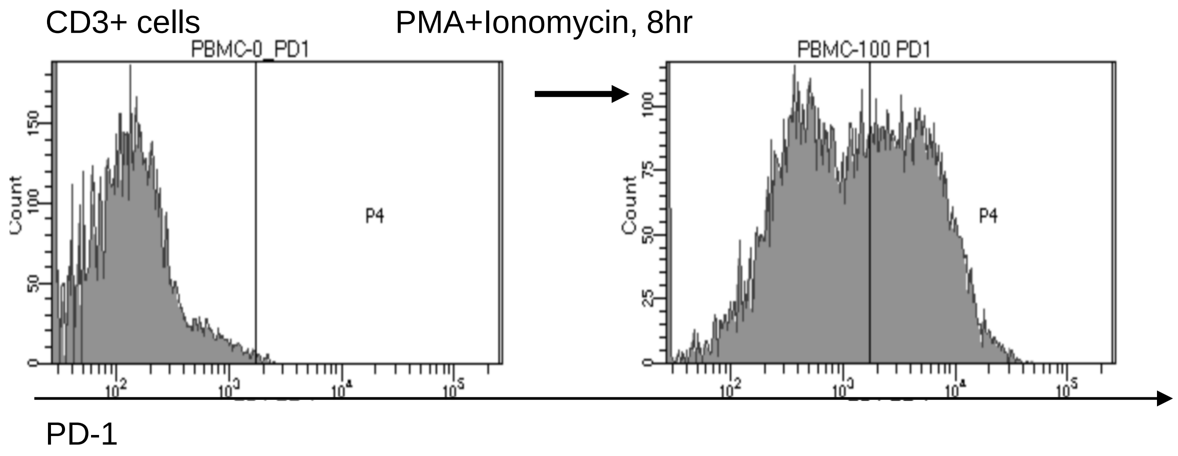

Supplementary Figure S6

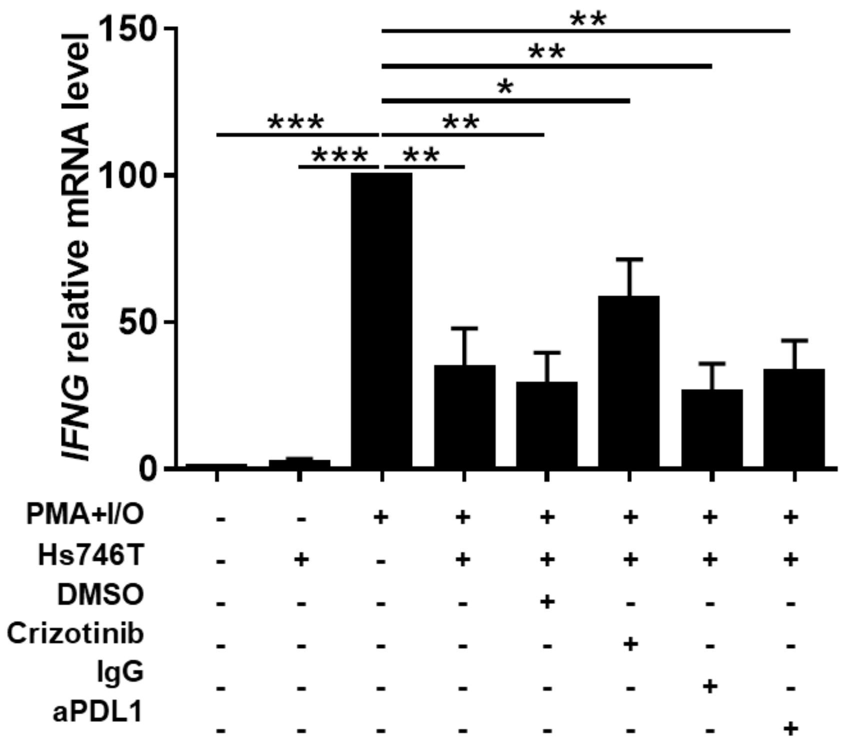

Supplementary Figure S7

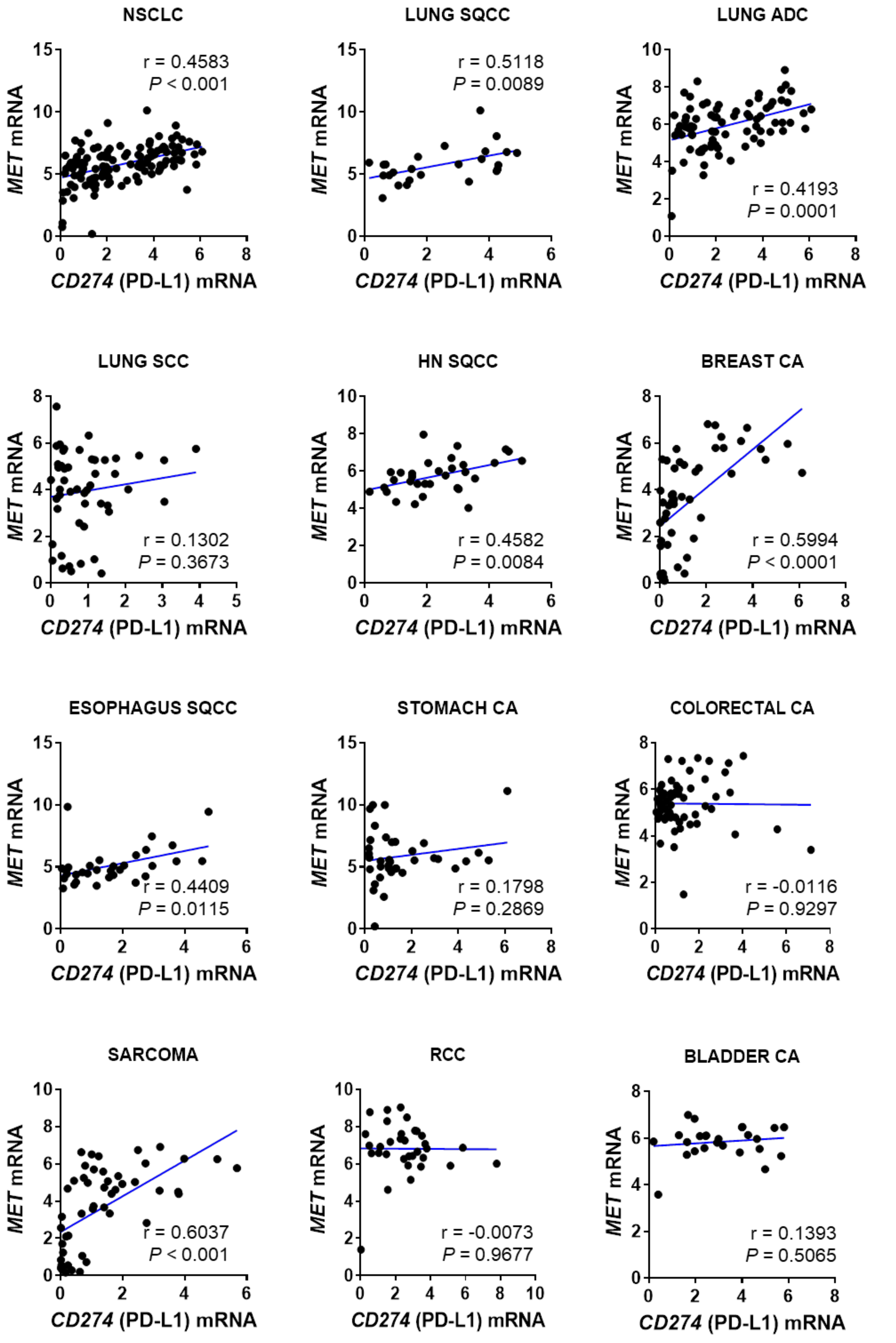

Supplement: Supplementary file 1 [file ijms-20-04287-s001.zip › ijms-566225 suppl for final/ijms-566225 Supplementary Figures_Revision 2.pdf]
